# Profiles of self-efficacy among cirrhotic patients in northern Guizhou Province, China: a latent profile analysis

**DOI:** 10.3389/fmed.2025.1627132

**Published:** 2025-07-10

**Authors:** Chengde Su, Qianqian Zhu, Mingdan Li, Yali Xu, Qian Liu, Ying Zhang, Xinyi Zhang, Huajun Wang, Qiuxiang Li, Ping Yang

**Affiliations:** ^1^Department of Infectious Diseases, Affiliated Hospital of Zunyi Medical University, Zunyi, Guizhou, China; ^2^Department of Nursing, Affiliated Hospital of Zunyi Medical University, Zunyi, Guizhou, China; ^3^School of Nursing, Zunyi Medical University, Zunyi, Guizhou, China

**Keywords:** liver cirrhosis, self-efficacy, self-management, latent profile analysis, influencing factors

## Abstract

**Objective:**

To explore the distinct profiles and influencing factors of self-efficacy in patients with liver cirrhosis via latent profile analysis (LPA) to provide evidence for the development of targeted interventions.

**Methods:**

This was a single-center, cross-sectional study in which convenience sampling was used to recruit hospitalized cirrhotic patients between March and November 2024 from the Department of Infectious Diseases of a tertiary general hospital in Zunyi, Guizhou Province. Data were collected via four validated instruments: the General Information Questionnaire, the Chronic Disease Management Self-Efficacy Scale, the Self-Management Behavioral Scale for Patients with Cirrhosis, and the Social Support Rating Scale. Latent profile analysis (LPA) Mplus 8.3 and univariate and multivariate logistic regression analyses were performed via SPSS 26.0.

**Results:**

A total of 260 questionnaires were distributed, with 257 valid responses collected, resulting in a response rate of 98.85%. Three distinct self-efficacy profiles were identified: low (7.39%), moderate (31.91%), and high (60.70%). Residence (OR = 0.055, 95% CI: 0.006–0.528), self-management score (OR = 0.846, 95% CI: 0.729–0.981), and social support score (OR = 0.655, 95% CI: 0.537–0.800) were significant predictors (*p* < 0.05).

**Conclusion:**

There was significant heterogeneity in the self-efficacy level of patients with liver cirrhosis. Healthcare professionals should provide targeted interventions addressing their specific needs on the basis of the distinct self-efficacy profiles of patients to increase self-efficacy levels and improve the quality of life of this population.

## 1 Introduction

Liver cirrhosis ranks as the 11th leading cause of death globally and the third most common cause of mortality among individuals aged 45–64 years (1). An estimated 2 million deaths worldwide are attributed to liver diseases annually, with 1 million directly linked to cirrhosis (1). As a severe clinical condition characterized by high morbidity, mortality, and fatality rates, cirrhosis is associated with a prolonged and recurrent disease course. These challenges not only increase the economic burden on patients and their families but also significantly impair their psychological well-being and quality of life, ultimately contributing to diminished self-efficacy among these patients (2). Studies highlight a robust association between self-management and self-efficacy (3, 4). However, cirrhosis patients often experience prolonged survival with multimorbidity and declining self-management capacity, leading to reduced treatment adherence and pervasive self-efficacy deficits—a critical determinant of disease prognosis (5, 6). Cirrhosis, a chronic and progressive liver disease, often presents with serious complications such as upper gastrointestinal bleeding, hepatic encephalopathy, and intractable ascites during the compensated phase (7). In severe cases, patients may develop both acute on chronic liver failure, resulting in clinical deterioration or poor therapeutic response (8). This can adversely affect their psychological well-being and reduce their sense of self-efficacy.

Self-efficacy, defined as an individual’s belief in their ability to execute specific behaviors to achieve desired outcomes (9), significantly influences emotional regulation, cognitive processing, and decision-making, particularly in adversity (10). In health psychology, self-efficacy is pivotal in shaping health behaviors, health literacy, and quality of life among chronic disease patients, serving as a key mediator influencing the efficacy of self-management (11). Notably, patients with greater self-efficacy demonstrate better disease management, greater adherence to therapeutic regimens, and greater perceived control over their lives (12). Current research on self-efficacy in patients with cirrhosis has predominantly employed a traditional variable-centered approach (13). This approach focuses on overall levels and influencing factors but overlooks the multidimensional nature of self-efficacy and the inherent heterogeneity within the patient population. Consequently, the ability to develop precise interventions is limited. Given that different cirrhotic patients experience distinct self-efficacy challenges, uniform intervention programs may be ineffective. There is a need for targeted interventions tailored to specific subgroups. Therefore, this study aims to utilize latent profile analysis to identify distinct profiles of self-efficacy in cirrhotic patients. This approach helps clarify the characteristics and influencing factors of self-efficacy across different subgroups, facilitating the development of targeted interventions.

Latent profile analysis (LPA) is a person-centered statistical method that identifies unobserved subgroups within a population on the basis of their patterns of responses across a set of continuous observed indicator variables (14). The model assumes conditional independence of the observed indicators within each latent profile. Importantly, this method allows for the identification of populations most in need of intervention and identifies where there is a need for intervention in different domains. Shao et al. (15) conducted a questionnaire survey on the self-management behaviors of elderly patients and classified the population into 3 categories through LPA, which revealed the heterogeneity of elderly patients with chronic diseases and facilitated the development of targeted interventions according to the different needs of elderly patients in the later stages of life to improve their quality of life. Traditional methods such as exploratory factor analysis and cluster analysis can identify the overall structure or group distribution, but it is difficult to reveal the intrinsic associations between different dimensions (16). In contrast, LPA is the preferred method for analyzing the heterogeneity of self-efficacy in cirrhosis patients because it can systematically characterize the combination of multidimensional self-efficacy in cirrhosis patients, quantify the probability of belonging to different efficacy profiles, and directly assess the synergistic effect among dimensions through model parameters.

The aim of this study was to categorize the self-efficacy categories of people with cirrhosis through LPA and to analyze the factors that influence them. We anticipate that the findings of this study may contribute to the development of tailored interventions on the basis of the identified patterns, especially for those with low levels of self-efficacy, to increase the level of self-efficacy in this population.

## 2 Methods

### 2.1 Design and participants

**Figure 1:**
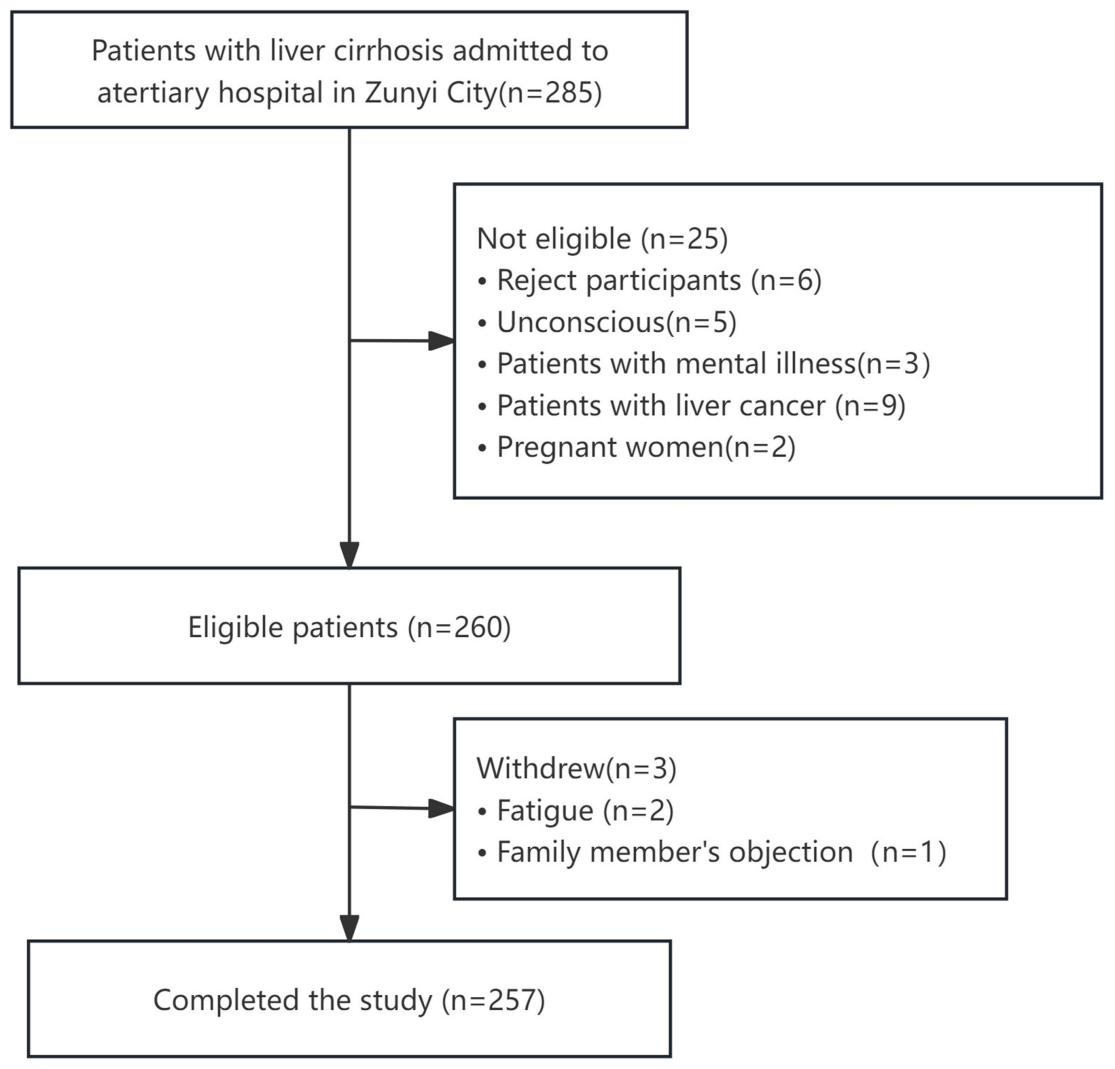
Flowchart of participant selection.

This single-center, cross-sectional study was conducted from March 2024 to November 2024 in the infection department of a tertiary general hospital in Zunyi, Guizhou Province. Before the questionnaires were distributed, the purpose, risks, and benefits of the study were explained verbally by trained nurses during room visits. Paper questionnaires were distributed to all participants who met the inclusion criteria and volunteered to participate. Among the 285 eligible patients, 260 (91.2%) gave initial consent; 3 withdrew because of fatigue (*n* = 2) or family opposition (*n* = 1) (see Figure 1). Missing item-level data (<5% per scale) were handled by multiple imputation using all-condition norms.

The inclusion criteria were as follows: (1) diagnosis of liver cirrhosis confirmed by the Guidelines for the Diagnosis and Treatment of Liver Cirrhosis (17); (2) age ≥18 years; (3) absence of cognitive, comprehension, or communication impairments; and (4) voluntary participation with signed informed consent. The exclusion criteria were as follows: (1) severe cognitive impairment, hepatic encephalopathy or other psychiatric disorders; and (2) comorbidities, including rheumatoid arthritis, malignancies, severe cardiovascular or cerebrovascular diseases, advanced osteoarthritis, or traumatic hand injuries. (3) refused to answer or provide incomplete responses to the questionnaire.

### 2.2 Sample size

It is generally suggested that the sample size for multivariate statistics should be more than 10 events per variable (18). In our study, the regression analysis included 18 observational variables, so the sample size should be a minimum of 180 people.

### 2.3 Measurements

#### 2.3.1 Sociodemographic and disease-related characteristics

The general information questionnaire was designed by the researcher and included age, sex, ethnicity, marital status, nature of work, education, place of residence, living situation, smoking history, drinking history, frequency of exercise, personal monthly income, degree of condition, Child–Pugh classification, and comorbidities.

#### 2.3.2 Self-efficacy to manage chronic disease scale (SEMCD-6)

The self-efficacy to manage chronic disease scale (SEMCD) was developed by the Center for Patient Education at Stanford University (19) and simplified to 6 items by Lorig et al. (20) (SEMCD-6). The SEMCD assesses patient confidence in managing fatigue, pain, mood, symptoms, activities, and medication management, with items 1 to 4 for symptom management and items 5 to 6 for disease comorbidity management. Each item is rated on a scale from 1 to 10. The mean score across the 6 items is calculated and categorized as low (≤4.0), moderate (4.0–7.9), or high (≥8.0) self-efficacy (20), with Cronbach’s alpha coefficients ranging from 0.88 to 0.95 and good internal consistency. The scale is widely used in the field of liver disease.

#### 2.3.3 Liver cirrhosis self-management behavior scale

This 24-item scale, adapted from Chinese and European clinical guidelines by Wang et al. (21), is used to evaluate self-management behaviors across four domains: diet, daily living, medication adherence, and disease monitoring. Each item was rated on a 4-point Likert scale (total score: 24--96), with higher scores indicating better self-management. The scale exhibited strong reliability and validity.

#### 2.3.4 Social support rating scale (SSRS)

The SSRS, revised by Xiao (22), measures social support across three dimensions: objective support, subjective support, and support utilization. The 10-item scale yields a total score of 66, categorized as low (<22), moderate (22–44), or high (>44) support. In this study, Cronbach’s *α* was 0.99.

### 2.4 Data collection and quality control

After approval from the head of the department was obtained, the researchers used a standardized script to explain the study to the participants and emphasized that they had the right to withdraw from the study at any time and that there would be no consequences for withdrawal. The questionnaires were then distributed to patients who agreed to participate, and patients were able to complete the questionnaires independently or receive assistance in completing them if needed. Electronic medical records were used to supplement disease-related information. All questionnaires were reviewed for completeness, and clarification was provided for any missing data. The questionnaires were considered invalid if more than 10% of the items were blank, had extreme values, or had multiple answers for multiple-choice items (23). To ensure the protection of participants’ privacy, all personally identifiable information, such as name and contact information, was deleted. All the data were stored securely and accessed only by authorized researchers through a password-protected electronic system.

### 2.5 Data analysis

LPAs were constructed via Mplus 8.3. Model fit was assessed via the following methods (24): log-likelihood (LL), Akaike information criterion (AIC), Bayesian information criterion (BIC), and sample-adjusted BIC (aBIC), with lower values indicating better fit; Lo–Mendell–Rubin (LMR) and bootstrap likelihood ratio tests (BLRTs), where *p* < 0.05 favored the k-class model over the (k-1)-class model; and entropy (0–1), with values ≥0.80 indicating high classification accuracy. SPSS 26.0 was used for descriptive statistics (frequency/percentage for categorical variables; mean ± SD for continuous variables). Chi-square tests, ANOVA, and multivariate logistic regression (*p* < 0.05) revealed differences in self-efficacy, self-management, and social support across latent profiles.

## 3 Results

### 3.1 Comparison of participants’ general information

A total of 260 questionnaires were distributed, and 257 valid questionnaires were recovered, with a valid questionnaire recovery rate of 98.85%. A total of 257 patients with liver cirrhosis were included in this study, and univariate analysis revealed that the differences in residence, exercise frequency (≥30 min/time), monthly personal income, spontaneous peritonitis, hepatic encephalopathy, ascites, Child–Pugh classification, total self-management score, and total social support score were statistically significant (*p* < 0.05), whereas the differences in other general information were not statistically significant (*p* > 0.05), as detailed in Table 1.

**Table 1:** Comparison of the general information of patients with cirrhosis in different self-efficacy categories (*n* = 257).

Characteristic	Categorization	Low self-efficacy (*n* = 19)	Moderate self-efficacy (*n* = 83)	High self-efficacy (*n* = 155)	*F*/χ^2^	*P*
Gender, *N*(%)	Male	12(63.2)	57(69.5)	101(64.7)	0.311	0.733
	Female	7(36.8)	25(30.5)	55(35.3)		
Age, mean ± SD	<60	14(73.7)	58(70.7)	125(80.1)	1.375	0.255
	≥60	5(26.3)	24(29.3)	31(19.9)		
Ethnic group, *N*(%)	Han ethnic	17(89.5)	69(84.1)	138(88.5)	0.492	0.612
	Minority ethnic	2(10.5)	13(15.9)	18(11.5)		
Marital status, *N*(%)	Married	19(100)	79(96.3)	150(96.2)	0.372	0.690
	Single/Divorced/Widowed	0(0)	3(3.7)	6(3.8)		
Nature of work, *N*(%)	Manual labor	12(63.2)	58(70.7)	90(57.7)	1.955	0.144
	Non-physical	7(36.8)	24(29.3)	66(42.3)		
Educational level, *N*(%)	High school and above	2(10.5)	11(13.4)	27(17.3)	0.504	0.605
	Junior high school and below	17(89.5)	71(86.6)	129(82.7)		
Place of residence, *N*(%)	Urban	2(10.5)	58(70.7)	99(63.5)	13.159	<0.001
	Rural	17(89.5)	24(29.3)	57(36.5)		
Living arrangement, *N*(%)	Living with a spouse	15(78.9)	61(74.4)	119(76.3)	0.299	0.742
	Living with children	2(10.5)	6(7.3)	10(6.4)		
	Living alone	2(10.5)	14(17.1)	16(10.3)		
	Living with others	0	1(1.2)	11(7.0)		
Smoking, *N*(%)	No	12(63.2)	40(48.8)	93(59.6)	1.472	0.231
	Yes	7(36.8)	42(51.2)	63(40.4)		
Drinking alcohol, *N*(%)	No	12(63.2)	53(64.6)	121(77.6)	2.708	0.069
	Yes	7(36.8)	29(35.4)	35(22.4)		
Exercise frequency (≥30 min/time), *N*(%)	<3 times/week	15(78.9)	50(61.0)	80(51.3)	3.186	0.043
	≥3 times/week	4(21.1)	32(39.0)	76(48.7)		
Monthly personal income (*$*), *N*(%)	<138	4(21.1)	21(25.6)	24(15.4)	3.557	0.030
	138–414	3(15.8)	22(26.8)	62(39.7)		
	414–690	7(36.8)	26(31.7)	47(30.1)		
	>690	5(26.3)	13(15.9)	23(14.7)		
Form of medical insurance, *N*(%)	Urban residents’ medical insurance	17(89.5)	74(90.2)	135(86.5)	0.367	0.693
	Employee medical insurance	2(10.5)	8(9.8)	21(13.5)		
Cirrhosis Stage, *N*(%)	Decompensated phase	15(78.9)	73(89.0)	129(82.7)	1.051	0.351
	Compensated phase	4(21.1)	9(11.0)	27(17.3)		
Complications						
Hypersplenism, *N*(%)	No	12(63.2)	58(70.7)	125(80.1)	2.215	0.111
	Yes	7(36.8)	24(29.3)	31(19.9)		
Spontaneous peritonitis, *N*(%)	No	16(84.2)	50(61.0)	120(76.9)	4.225	0.016
	Yes	3(15.8)	32(39.0)	36(23.1)		
Hepatic encephalopathy, *N*(%)	No	11(57.9)	54(65.9)	123(78.8)	3.583	0.029
	Yes	8(42.1)	28(34.1)	33(21.2)		
Ascites, *N*(%)	No	13(68.4)	47(57.3)	115(73.7)	3.376	0.036
	Yes	6(31.6)	35(42.7)	41(26.3)		
Hepatorenal syndrome, *N*(%)	No	18(94.7)	70(85.4)	139(89.1)	0.768	0.465
	Yes	1(5.3)	12(14.6)	17(10.9)		
Electrolyte disorders, *N*(%)	No	14(73.7)	71(86.6)	139(89.1)	1.821	0.164
	Yes	5(26.3)	11(13.4)	17(10.9)		
Child–Pugh class, *N*(%)	Grade A + B	14(73.7)	47(57.3)	116(74.4)	3.819	0.023
	Grade C	5(26.3)	35(42.7)	40(25.6)		
Self-management score (points, mean ± SD)	79.66 ± 0.461	64.79 ± 2.037	77.38 ± 0.615	82.67 ± 0.407	96.579	<0.001
Social support score (mean ± SD)	43.82 ± 0.454	30.68 ± 1.300	40.09 ± 0.746	47.38 ± 0.346	113.356	<0.001

### 3.2 LPA of self-efficacy profiles in liver cirrhosis patients

Exploratory LPA was conducted with 1- to 4-class models to identify optimal self-efficacy profiles (Table 2). The number of model categories increased from 1 to 4, and the AIC, BIC and aBIC continued to decrease, with the entropy being highest in the 2-profile model. Four profile models with LMR and BLRT had *p*-values >0.05 and were excluded. The 3-profile model demonstrated lower AIC, BIC, and aBIC values than did the 2-profile model. It also had the lowest BIC, acceptable entropy (>0.80), and statistically significant LMRT and BLRT (*p* < 0.05), leading to its selection as the optimal profile solution.

**Table 2:** Fitted indicators of potential profiles of self-efficacy in patients with cirrhosis (*n* = 257).

Profile	AIC	BIC	aBIC	Entropy	LMR (*P*)	BLRT (*P*)	Class counts
1	4595.316	4637.905	4599.862	–	–	–	100
2	4078.017	4145.45	4085.214	0.959	0.0004	0.0005	19.455/80.545
3	3829.034	3921.310	3838.883	0.947	0.0011	0.0012	7.393/31.907/60.7
4	3714.635	3831.754	3727.134	0.932	0.266	0.2739	6.226/31.518/34.241/28.016

**Figure 2:**
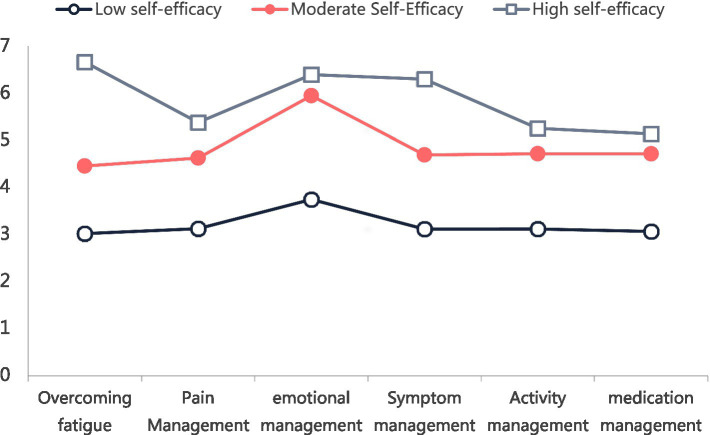
Distribution of 3 potential profile characteristics of self-efficacy in cirrhotic patients.

Figure 2 shows the mean scores across the six SEMCD-6 dimensions for the three latent profiles. On the basis of their distinct response patterns, Class 1 (low self-efficacy; *n* = 19, 7.39%) patients presented uniformly low scores across all dimensions, particularly in symptom management. Class 2 (moderate self-efficacy; *n* = 83, 31.91%) was characterized by intermediate scores with notable deficits in symptom management. Class 3 (High Self-Efficacy; *n* = 155, 60.70%) achieved the highest total and dimension-specific scores and excelled in symptom management.

### 3.3 Comparative analysis of self-efficacy scores across latent profiles

Significant differences (*p* < 0.05) were observed in total self-efficacy scores and all subdimensions among the three latent profiles (Table 3).

**Table 3:** Scores of patients with cirrhosis in different potential profiles (score, mean ± SD).

Scores for each dimension of self-efficacy	Total number of cases (*n* = 257)	Low self-efficacy (*n* = 19)	Moderate self-efficacy (*n* = 82)	High self-efficacy (*n* = 156)	*F*	*P*
Overcoming Fatigue	5.67 ± 1.514	3 ± 0.577	4.43 ± 0.609	6.65 ± 0.941	302.669	<0.001
Pain Management	4.96 ± 1.049	3.11 ± 0.658	4.62 ± 0.601	5.37 ± 0.964	70.329	<0.001
Emotional Management	6.05 ± 1.022	3.74 ± 0.733	5.94 ± 0.691	6.38 ± 0.799	103.579	<0.001
Symptom Management	5.54 ± 1.149	3.11 ± 0.737	4.66 ± 0.477	6.29 ± 0.535	463.566	<0.001
Activity Management	4.91 ± 0.875	3.11 ± 0.809	4.72 ± 0.479	5.24 ± 0.737	90.408	<0.001
Medication Management	5.13 ± 0.825	3.05 ± 0.705	4.71 ± 0.509	5.13 ± 0.825	70.463	<0.001
Chronic Disease Management Self-Efficacy Scale Total Score	32.00 ± 5.130	19.11 ± 3.478	29.2 ± 2.344	32 ± 5.130	446.29	<0.001

### 3.4 Univariate analysis of latent self-efficacy profiles

The three latent profiles significantly differed in residence location, exercise frequency, monthly income, complications (spontaneous bacterial peritonitis, hepatic encephalopathy, ascites), and Child–Pugh classification (*p* < 0.05; Table 1).

### 3.5 Multivariate logistic regression analysis

Variables with significant univariate associations were included as predictors in a multivariate logistic regression model (reference: high self-efficacy). Independent variables were coded as detailed in Table 4. The results revealed that residence (OR = 0.055, 95% CI: 0.006–0.528), self-management score (OR = 0.846, 95% CI: 0.729–0.981), and social support score (OR = 0.655, 95% CI: 0.537–0.800) were independent predictors of latent profile membership (*p* < 0.05; Table 5), and multicollinearity diagnostics, as detailed in Table 6.

**Table 4:** Independent variable assignment methods.

Independent variable	Assignment method
Residence	Urban = 1; Rural = 2
Monthly Personal Income	<1,000 = 1; 1,000–3,000 = 2; 3,001–5,000 = 3; >5,000 = 4
Exercise frequency	<3 times/week = 1; ≥3 times/week = 2
Spontaneous peritonitis	None = 0; Yes = 1
Hepatic encephalopathy	None = 0; Yes = 1
Ascites	None = 0; Yes = 1
Child–Pugh Score	Class A + Class B = 1; Class C = 2
Self-management score	Original value input
Social Support Score	Original value input

**Table 5:** Logistic regression analysis of factors influencing potential categories of self-efficacy in cirrhotic patients.

Profile	Variables	*β*	Standard error	*P*	*OR*	95%*CI*
Low self-efficacy^a^	Intercept	26.452	5.045	<0.001		
	Residence in urban areas	−2.893	1.151	0.012	0.055	0.006–0.528
	Total Social Support Score	−0.423	0.102	<0.001	0.655	0.537–0.800
	Total self-management score	−0.168	0.076	0.027	0.846	0.729–0.981
Moderate self-efficacy^b^	Intercept	−7.813	3.869	0.043		
	Residence in urban areas	2.811	1.109	0.011	16.619	1.890–146.099
	Total Social Support Score	0.211	0.095	0.026	1.235	1.025–1.488
High self-efficacy^c^	Intercept	−18.640	3.290	<0.001		
	Total Social Support Score	0.211	0.041	<0.001	1.235	1.140–1.338
	Total self-management score	0.111	0.038	0.004	1.118	1.037–1.205

^a^Reference group: high self-efficacy; ^b^reference group: low self-efficacy; ^c^reference group: moderate self-efficacy.

**Table 6:** Multicollinearity diagnostics.

Class	Variables	OR(95% CI)	*β*	VIF	*P*
Class 1	Total Social Support Score	0.059(0.052,0.067)	0.685	1.000	<0.001
Class 2	Total Social Support Score	0.041(0.031,0.051)	0.476	1.796	<0.001
	Total self-management score	0.027(0.017,0.036)	0.313	1.796	<0.001
Class 3	Total Social Support Score	0.042(0.032,0.052)	0.485	1.803	<0.001
	Total self-management score	0.025(0.015,0.035)	0.294	1.826	<0.001
	Residence (reference: urban)	−0.139(−0.249,−0.028)	−0.107	1.018	0.014

## 4 Discussion

### 4.1 Heterogeneity in the self-efficacy profiles of liver cirrhosis patients

The results of this study revealed that there was a significant heterogeneous characterization of self-efficacy in patients with cirrhosis, mainly in the low self-efficacy group, moderate self-efficacy group and high self-efficacy group. The total score of the self-efficacy assessment scale in cirrhotic patients was 32.00 ± 5.13, which was moderate and was consistent with the results of a previous study (25). This may be related to the fact that patients with cirrhosis suffer from chronic disease symptoms, recurrent hospital admissions and continuous medical expenditures, leading to a decrease in their self-efficacy (26). Within the low self-efficacy group (7.39%), scores on the emotion management dimension were relatively higher than those on other dimensions, which may be attributed to the current medical technology’s emphasis on psychological interventions for patients (27); however, other dimensions were low, suggesting that this group of patients still had greater difficulties with other aspects of self-efficacy, especially in disease management and treatment perceptions, which may be related to cirrhotic patients’ general lack of adequate knowledge of treatment modalities. The low score on the symptom management dimension in the moderate self-efficacy group (31.91%) suggests that patients are deficient in cirrhosis symptom recognition and management strategies. Patients’ lack of symptom recognition may affect their treatment adherence and disease control, so there is a need to enhance patients’ symptom management education and improve their self-management skills. The high self-efficacy group (60.70%) had better overall dimensions, which may be related to their higher health literacy and accessibility of urban medical resources. Therefore, healthcare professionals should not only pay attention to the level of self-efficacy of cirrhotic patients but also identify their potential categories and construct a stepwise intervention strategy: for low self-efficacy, multidimensional structured interventions should be adopted to improve the ability of cirrhotic patients in the comprehensive management of the disease; for the moderate self-efficacy group, goal-oriented supportive interventions should be adopted to broaden the patient’s knowledge of the symptoms of the disease and improve the adherence of the patients; for the high self-efficacy group, maintenance self-management should be carried out.

### 4.2 Determinants of self-efficacy heterogeneity

#### 4.2.1 Cirrhotic patients living in rural areas are more likely to be categorized into the low self-efficacy group

A global burden of disease estimates (28) that there are approximately 112 million patients with compensated cirrhosis worldwide and that the burden of disease is influenced by a variety of factors, such as the living environment, healthcare system, ethnicity, quality of education, and socioeconomic status (29). This difference in health outcomes due to differences in geographic and social environments presents significant manifestations in the level of patient self-efficacy. The results of this study revealed that the risk attributed to low self-efficacy in cirrhotic patients residing in rural areas was 16.62 times greater than that attributed to moderate self-efficacy, which is consistent with the findings of Ran and Hu (30). The reasons for this difference are closely related to the differences in health resources, medical services, social support and health education between urban and rural areas (31). In a study by He et al. (32), 440 adolescent HIV patients were investigated, and patients living in cities and towns had higher levels of self-efficacy, which may be attributed to the fact that adolescent patients living in urban areas had more opportunities for exposure to sexual safety education at an early age, which reflects the positive impact of urban environments on individuals’ health coping ability. Urban areas are rich in health resources, medical services, and educational opportunities, which help residents improve their self-management skills and thus enhance their self-efficacy (33). The participants in this study were mainly from economically underdeveloped rural or urban areas in Southwest China. However, remote areas face a shortage of healthcare resources, with a limited number and uneven distribution of healthcare professionals (15). This lack of resources results in fewer chronic disease health education programs, limits patients’ access to timely and effective medical care, and restricts opportunities for doctor–patient communication. Despite China’s progress in achieving universal health coverage, limited economic development, health resource allocation, and population health status among rural households in western China have led to inadequate health perceptions among residents, a lack of confidence in coping with challenges, and reduced self-efficacy (34). This disparity highlights the critical impact of the ‘digital divide’ in healthcare access and information. Our proposed strategies, such as leveraging short videos for evidence-based science popularization and digital health interventions through grassroots health centers, aim to bridge this gap by delivering essential knowledge and support remotely.

#### 4.2.2 Cirrhotic patients with higher levels of social support were more likely to be categorized into the high self-efficacy group

The results of this study revealed that the level of social support was positively correlated with self-efficacy, which is consistent with the findings of Kleppang et al. (35). According to related studies (36), the level of social support, which usually comes from social support networks such as family, friends, and social networking platforms, has a significant effect on self-efficacy in patients with cirrhosis and is significantly associated with health literacy and quality of life. Cirrhotic patients with lower levels of social support are more likely to engage in alcohol abuse, smoking, poor diet, and poor treatment adherence, which not only exacerbates the pathological process of liver injury but also weakens self-efficacy, forming a vicious cycle (37). Domestic scholars have conducted a cross-sectional study of 6,075 adolescents (38), and the results revealed a significant association between high levels of social support and higher self-efficacy, a finding that further validates the important influence of social support on individual health behaviors. In patients with cirrhosis, adequate social support can significantly increase self-efficacy, help patients better manage their disease, improve treatment adherence, effectively promote patients’ active participation in social activities, increase interpersonal interactions, and reduce isolation caused by the disease, thus significantly improving quality of life and social functioning (34). Therefore, it is recommended that medical professionals encourage family members to participate in the treatment and management of patients, especially in terms of emotional and instrumental support. Combined with telemedicine technology, online health counseling and monitoring can be provided to broaden the scope of support. Moreover, a community platform is established to promote patient experience sharing and mutual support to further enhance self-efficacy and treatment outcomes.

#### 4.2.3 Cirrhotic patients with higher levels of self-management were more likely to be categorized into the high self-efficacy group

Self-management refers to an individual’s ability to manage symptoms, treatments, physical and psychosocial consequences, and lifestyle changes inherent to living with a chronic disease and directly affects an individual’s ability to manage their health and daily life proactively (3, 4). The results of this study revealed that cirrhotic patients with high total self-management scores were more likely to attribute high self-efficacy, similar to the findings of Wang et al. (25), highlighting self-management as a key correlate and potential driver of self-efficacy. Iranian scholars conducted a clinical trial on 80 patients with liver cirrhosis and reported that the total self-efficacy score of the intervention group receiving self-management training was significantly higher than that of the control group (39). Zhang et al. (34) reported that standardized self-management intervention programs not only effectively reduce the economic burden on patients and improve their quality of life but also directly increase self-efficacy by strengthening individuals’ sense of control over the disease (39). Enhancing self-efficacy self-management can help patients accumulate coping experience and enhance their confidence in disease control by providing positive feedback, increasing control, reducing anxiety, and improving coping strategies, thus improving self-efficacy and optimizing the effectiveness of health management and treatment (12, 40). Therefore, clinical staff should enhance patients’ self-efficacy through health education and personalized guidance, encourage them to actively participate in treatment and care, improve their negative moods, and enhance their self-confidence to improve disease management and quality of life. Moreover, sentinel symptom monitoring techniques are utilized to identify disease changes in a timely manner and intervene in advance.

### 4.3 Limitations

This study has several limitations. First, the use of convenient sampling from a single tertiary care hospital in Zunyi, China, limits the generalizability of the findings. The geographic and socioeconomic context of Southwest China may not represent cirrhosis patients in other regions or countries. Second, the small sample size within the low self-efficacy profile (*n* = 19) reduced the statistical power for analyzing factors specific to this vulnerable subgroup and limits the generalizability of findings for this group. Third, some potentially relevant clinical covariates were not collected, which might influence self-efficacy profiles. Future research should employ multicenter designs with larger, stratified samples to validate these latent profiles across diverse settings. Finally, the cross-sectional design precludes establishing causal relationships or tracking dynamic changes in self-efficacy over time. Longitudinal studies are needed to confirm potential causal pathways and understand the evolution of self-efficacy profiles throughout the disease course.

## 5 Conclusion

By employing LPA, this study identified three distinct self-efficacy profiles among cirrhotic patients: low, moderate, and high. Significant differences were observed across these profiles in terms of place of residence, level of social support, and level of self-management. Our findings underscore the necessity for tailored, profile-specific interventions, particularly those focused on supporting those in the low self-efficacy profile, to effectively enhance self-management capabilities and ultimately improve quality of life in this patient population.

## Data Availability

The original contributions presented in the study are included in the article/supplementary material, further inquiries can be directed to the corresponding author.
